# Comparison of Phytochemical Profile and In Vitro Bioactivity of Beverages Based on the Unprocessed and Extruded Sesame (*Sesamum indicum* L.) Seed Byproduct

**DOI:** 10.3390/foods11203175

**Published:** 2022-10-12

**Authors:** María Fernanda Quintero-Soto, Ramona Julieta Espinoza-Moreno, Jennifer Vianey Félix-Medina, Fernando Salas-López, Cruz Fernando López-Carrera, Oscar Daniel Argüelles-López, Martha Elena Vazquez-Ontiveros, Mario Armando Gómez-Favela

**Affiliations:** 1 Ingeniería Agroindustrial, Universidad Politécnica del Mar y la Sierra (UPMYS), La Cruz, Elota 82700, Mexico; 2 Ingeniería Bioquímica, Tecnológico Nacional de México-Instituto Tecnológico de Culiacán, Culiacan 80220, Mexico; 3 Licenciatura en Ciencias Biomédicas, UR Culiacán, Universidad Autónoma de Occidente (UAdeO), Culiacan 80020, Mexico; 4 Escuela Nacional de Ciencias Biológicas, Instituto Politécnico Nacional (IPN), Mexico City 11340, Mexico; 5 Facultad de Ciencias Químico-Biológicas, Universidad Autónoma de Sinaloa (UAS), Culiacan 80010, Mexico

**Keywords:** dipeptidyl-peptidase-IV, α-amylase, α-glucosidase, HPLC-MS/MS, Melanoidins

## Abstract

In this research functional beverages based on the unprocessed and extruded sesame seeds byproduct were fabricated; phytochemical profile, antioxidant, antidiabetic, and hypoglycemic potential were evaluated. Twenty-four phytochemical compounds were identified in total in both beverages; fourteen of the phytochemical compounds were not modified by the extrusion process. Seventeen of the 24 compounds were identified in the unprocessed sesame seeds byproduct flour beverage−10% (UB10) and 21 in the extruded sesame seeds byproduct flour beverage−10% (EB10). The compounds only identified in UB10 are caffeic acid, luteolin-7-O-glucoside, and isorhamnetin; and in EB10 those compounds were vanillic acid, acteoside, luteolin, quercetin, and melanoidins. No significant difference was observed in the content of total phenolic compounds (TPC) (14.90 and 15.97 mg GAE/100 mL) and total flavonoids (TF) (5.37 and 5.85 mg QE/100 mL). An increase in the biological activity of ESFB10 (IC50: ABTS = 0.19, DPPH = 0.21, α-amylase = 1.01, α-glucosidase = 0.17, DPP4 = 0.11 mg/mL) was observed, compared to UB10 (IC50: ABTS = 0.24, DPPH = 0.31, α-amylase = 2.29, α-glucosidase = 0.47, DPP4 = 0.30 mg/mL). Therefore, the extrusion process had a positive effect, which displayed the highest efficiency inhibiting the free radicals and enzymes related to carbohydrate metabolism.

## 1. Introduction

Sesame seed (*Sesamum indicum* L.) is rich in protein (17.7%), carbohydrates (23.4%), and, mainly, lipids (49.7%) [1]. In addition, it is rich in fatty acids (oleic, linoleic, palmitic, and stearic), minerals (iron, magnesium, manganese, copper, and calcium), and vitamins (B1 and E) [2]. Sesame seed also contains chemical compounds which, in excess, are known as antinutritional (phytic acid, oxalic acid, saponins, trypsin inhibitors, and lectins) because they hinder or inhibit the absorption of nutrients that come from food [3,4]. However, it has been reported that the content of these compounds can decrease by subjecting the seeds or flour undergo various processes [4,5,6,7].

Traditionally, sesame seeds are used in the formulation of paints, soaps, cosmetics, perfumes, pharmaceutical products, and, to a lesser extent, in the confectionery and baking industry [8,9]. However, sesame is mainly used for oil extraction due to its high content, which limits its consumption and usefulness, generating a large amount of sesame byproduct [10,11]. Rama et al. [11] estimate that 1.53 million tons of sesame byproduct per year are obtained worldwide. In addition, the sesame byproduct generated by oil extraction has many bioactivities that make it a candidate to be used as a raw material in the production of functional foods [12,13].

The demand for flavored beverages has increased worldwide in recent years; however, they are mostly made of sugar, making them high-energy-density beverages (hypercaloric) [14]. Modern consumers currently use functional foods that provide beneficial health effects, in addition to nutrient value and sensory pleasure. Based on this trend, a wide variety of beverages with low caloric intake have been developed; an example is low-sugar soft soda. [15]. Therefore, sesame seeds are an excellent option for elaborate functional beverages with beneficial health effects [15,16,17]. Sesame seed has been reported to have antioxidant, hypoglycemic, and anti-inflammatory effects, among others [18,19,20,21]. Despite the benefits of sesame seeds, the commercial use of this food has been limited. Hence the importance of developing alternatives technologies that allow healthy foods or beverages made from whole grains with health benefits [16,18,22,23].

Extrusion belongs to green processing technologies because it does not generate polluting effluents and offers numerous advantages, such as high productivity, high energy efficiency, and low operating costs, in addition to the high quality of the resulting products and more retention of bioactive compounds [7]. Several researchers have studied the extrusion process’s versatility for producing functional beverages. For example, Rochín–Medina et al. [14] fabricated an antioxidant beverage based on extruded corn-chickpea flours, and Argüelles–López et al. [24] developed an antioxidant and antihypertensive beverage from extruded amaranth–chia flour. However, a report on the analysis of the profile of phenolic compounds, antioxidant, hypoglycemic, and antidiabetic activities of extruded sesame flour beverages is not available. Therefore, the objective of this study was to fabricate a functional beverage based on extruded sesame flour and to evaluate its phenolic profile and the antioxidant, antidiabetic, and hypoglycemic in vitro potential.

## 2. Materials and Methods

### 2.1. Samples

Sesame seeds (*Sesamum indicum* L.) cultivated in the community of El Saladito, Elota, Sinaloa were used. The seeds were grown during the 2019 season.

### 2.2. Preparation of Optimized Extruded Sesame Flour

The extruded sesame seeds byproduct flour (SSBPF) was fabricated following the methodology optimized by Ruiz-Armenta et al. [25]. First, the sesame seeds byproduct was passed through a mechanical press twice to remove as much oil as possible. Next, the SSBPF obtained was conditioned with distilled water until reaching a moisture percentage of 23%. The extrusion was carried out on a single screw extruder model 20DN (CW Brabender Instruments, Inc., South Hackensack, NJ, USA) with a screw diameter of 19 mm; a length-to-diameter ratio of 20:1; compression ratio of 1:1, and an output die of 3 mm. The extrusion temperature and screw speed conditions used were TE = 139 °C/VT = 80 rpm. The extruded product was collected in trays and letting dry until reaching a water activity of 0.5 to prevent microbial growth. Finally, the pellets were ground, and the flour obtained was passed through sieve #100.

### 2.3. Preparation of the Beverages

The formulations to make the extruded sesame seeds byproduct flour beverage were made according to Rochín–Medina et al. [14] with slight modifications. Six formulations were prepared with different concentrations of extruded SSBPF (5, 10, 15, 20, 25, and 30%) and 5 g of sugar substitute (BC Metco Sugar (mixture of sucrose, high-intensity sweeteners, inulin, and fructo-oligosaccharides)). Purified water was added to the mixtures at room temperature (25 °C) to obtain a volume of 200 mL. The samples were stirred at 500 rpm/20 min and stored at 8 °C overnight (16 h). A total of 10 L of each formulation were prepared.

A beverage based on unprocessed SSBPF was also prepared. The same procedure was followed for the extruded flour beverage, replacing only the extruded flour by raw flour.

### 2.4. Sensory Evaluation of the Beverages

A sensory evaluation was performed on beverages to know their acceptability. One-hundred non-trained panelists (48 females and 52 males), in an age range of 18–56 years, no food allergies and willingness to participate were used. All beverages’ formulations were enumerated and presented tach evaluator in a completely random order. A commercial beverage (beverage purchased at a local store and made from barley flour, sugar, vanilla flavoring, cinnamon flavoring, and water) was used as control. Drinking water was provided to cleanse the palate between each sample. A descriptive analysis was performed on the samples: the evaluators qualified flavor, color, smell, and global acceptability. The acceptance level was determined using a bidirectional LAM scale with transformed values from zero to one hundred (0 = maximum imaginable rejection value, 100 = maximum acceptance value; 50 = neither likes nor dislikes) to evaluate the taste, color, aroma, and global acceptance to the beverages [26].

### 2.5. Extraction and Quantification of Phytochemicals from the Beverages

The selected beverage was freeze-dried and used to prepare phytochemical extracts, as described by Quintero–Soto et al. [27]. One gram of sample was homogenized (60 min/300 rpm) with 80% methanol (30 mL), hydrolyzed with 12 mL 2N HCl (30 min/90 °C), and centrifuged (10,000 g/30 min); the supernatant was mixed with hexane (40 mL) to remove fats. Subsequently, the aqueous phase was mixed with equal parts of deionized water and ethyl acetate. Ethyl acetate was recovered and taken to dryness. Finally, the dried samples were reconstituted in 1 mL of 80% methanol and stored at −20 °C until further use.

Methanolic extracts were used to determine the content of total phenolic compounds (TPC) and total flavonoids (TF) [28,29]. TPC were determined by mixing 200 μL of extract and 2.2 mL of Folin–Ciocalte reagent. The samples were allowed to stand for 3 min in the dark. Subsequently, 7% sodium carbonate (600 μL) was added and incubated in the dark at room temperature (90 min). Finally, the absorbance of the samples at 750 nm was measured and the results were expressed in mg gallic acid equivalents (mg GAE). To determine the TF content, 100 μL of extract, 500 μL of water, and 25 μL of 5% sodium nitrite were mixed. Samples were left standing for 6 min in the dark and 50 μL of 10% silver chloride was added. Subsequently, 250 μL of 1M sodium hydroxide was added. A wavelength around 510 nm was used to read the samples, and the results were expressed in mg of quercetin equivalents (mg QE). A commercial beverage (beverage purchased at a local store and made from barley flour, sugar, vanilla flavoring, cinnamon flavoring, and water) was used for compared the phytochemical content and bioactivities.

The separation and identification of the individual phenolic compounds were carried out following the methodology reported by Quintero–Soto et al. [27]. Twenty microliters of extract were injected into a HPLC-DAD (1100 Series, Agilent Technologies, Santa Clara, CA, USA) coupled to a mass detector (1100 Series LC/MSD Trap, Agilent Technologies, USA) and separated on a Zorbax SB-C18 column (150 × 3 mm, 5 μm particle size, Agilent Technologies, Santa Clara, CA, USA) using 99% water/1% formic acid (A) and acetonitrile (B) as mobile phases, and a linear gradient from 1 to 60% B in 60 min at 0.4 mL/min. The detection of the compounds was carried out at 280, 320, and 350 nm. The compounds were identified by operating in positive/negative mode (35 V, 300 °C) and the ions were fragmented via collision induced dissociation. Helium and nitrogen were used for the collision and drying, respectively. Data was analyzed with the MstReNova software and full scan spectra were acquired in the *m*/*z* range of 100 to 2000. The individual phenolic compound content was calculated using a standard curve of each external standard.

### 2.6. Determination of the Antioxidant, Hypoglycemic and Antidiabetic Activities

For the determination of in vitro antioxidant activity (AA), the ABTS and DPPH colorimetric methods were used [30,31]. The ABTS radical solution (7.4 mmol/L) was prepared by mixing of ABTS and potassium persulfate (2.6 mmol/L), followed by overnight incubation (16 h) in the dark at room temperature. The ABTS radical solution was diluted with phosphate buffer (10 mmol/L, pH 7.4) to obtain an absorbance of 0.70 at 734 nm. The solution (3 mL) was mixed with 0.75 mL of sample and incubated at room temperature for 6 min before reading at 734 nm. For DPPH, the sample (0.2 mL) was mixed with 1.8 mL of DPPH radical solution (0.1 mmol/L in ethanol) and incubated at room temperature for 30 min before reading at 510 nm. Both assays were performed in the dark.

The hypoglycemic activity was determined by measuring the inhibition of the *α*-amylase and *α*-glucosidase enzymes following the methodology proposed by Amutha and Godavari [18]. Equal parts of the sample and *α*-amylase solution (2 U/mL) were mixed and incubated (10 min, 37 °C), followed by the addition of starch solution (0.5 mL, 1%) and incubation (5 min, 37 °C). Subsequently, the sample was placed in a water bath (100 °C, 10 min) in the presence of dinitrosalicylic acid (1.6 mL). Finally, the sample was diluted with distilled water (2.5 mL), and the absorbance was reading at 540 nm. For *α*-glucosidase, the *α*-glucosidase solution (0.05 mL, 5 U/mL) y 0.5 mL of sample were mixed and incubated (10 min, 37 °C). Subsequently, p-nitrophenyl-*α*-*D*-glucopyranoside (0.5 mL, 5 mmol/L) was added and the mixture was incubated (10 min, 37 °C) before reading at 405 nm. Acarbose was used as a positive control.

The antidiabetic activity was determined by inhibiting the enzyme dipeptidyl-peptidase-IV (DPP4) using the MAK203 kit (Sigma-Aldrich, St. Louis, MO, USA). Sitagliptin was used as a positive control.

### 2.7. Statistical Analysis

The statistical analysis was performed in the software of STATGRAPHIC plus version 5.1 (Statistical Graphics Corporation, Rockville, MD, USA). The comparison of means was made using Fisher’s test and a significance level of 5%. Principal component analysis was performed to investigate the grouping and relationship of the extrusion process, metabolites, and bioactivities using the open-source R studio program version 3.6.2 (The R Foundation for Statistical Computing, Vienna, Austira).

## 3. Results and Discussion

### 3.1. Sensory Analysis of Beverage

**Figure 1:**
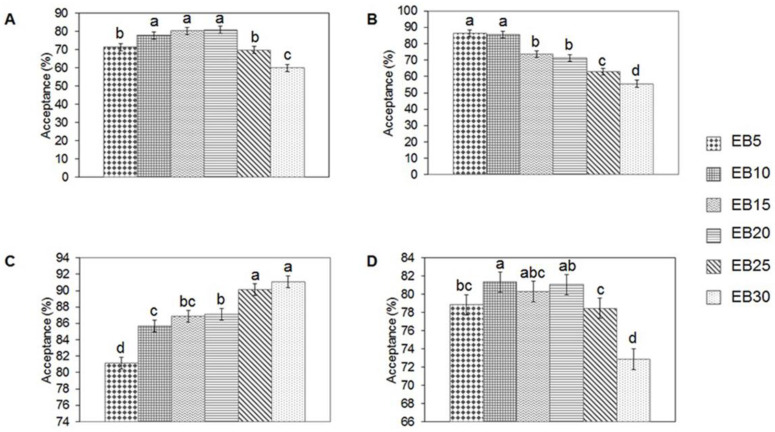
Sensory acceptance analysis of extruded sesame seeds byproduct flour beverages. (**A**) Flavor; (**B**) Color; (**C**) Smell; (**D**) Global Acceptability. EB5: Beverage with 5% extruded sesame seeds byproduct flour; EB10: Beverage with 10% extruded sesame seeds byproduct flour; EB15: Beverage with 15% extruded sesame seeds byproduct flour; EB20: Beverage with 20% extruded sesame seeds byproduct flour; BE25: Beverage with 25% extruded sesame seeds byproduct flour; BE30: Beverage with 30% extruded sesame seeds byproduct flour. Different letters in the same graph indicate significant differences (*p* < 0.05) among the means according to the Fisher’s test.

The beverage made with 10% extruded SSBPF showed the highest global acceptance by the panelists (Figure 1). Regarding taste, no significant difference was observed between beverages of 10, 15, and 20% (*p* < 0.05). Beverages colors ranged from light beige (5%) to dark beige (30%). A decrease in acceptance was observed as the concentration of SSBPF and the intensity of the beverage color increased. The smell of the beverages was more accepted when the concentration of extruded SSBPF was increased. On its own, extruded SSBPF smells like baked pastry products. According to the results obtained in the sensory analysis, the beverage with a concentration of 10% of extruded SSBPF was selected for subsequent analysis. This beverage provides 359.824 J/200 mL, so it follows the recommendations of the Ministry of Health and Assistance of Mexico, which indicate that a beverage of 200 mL should not contain more than 418.40 J (NMX-F-439-1983). This concentration of SSBPF (10%) had already been used in the preparation of cookies by Lucini Mas et al. [32]. They reported that this concentration had the best accepted by the panelists too.

### 3.2. Changes in Phytochemicals Profile Caused by the Extrusion Process

**Figure 2:**
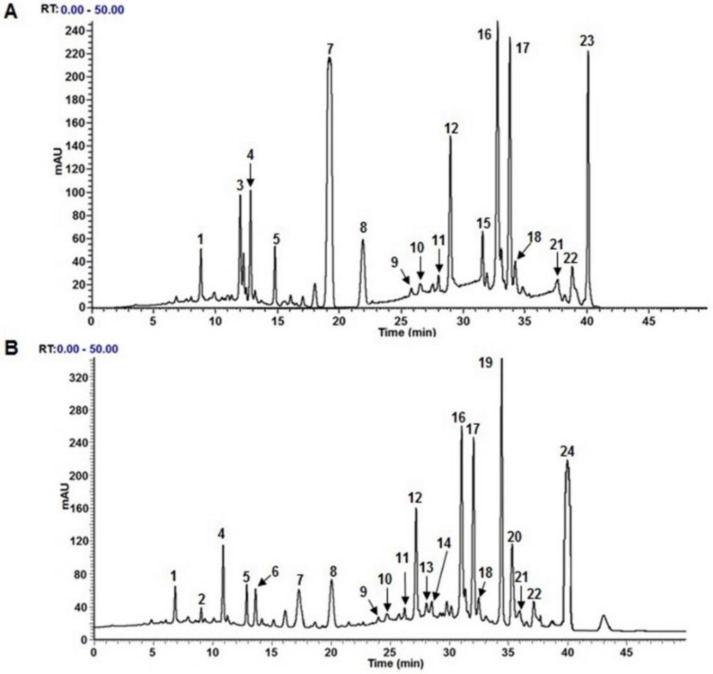
Chromatography separation of phytochemical compounds found in extracts of (**A**) unprocessed sesame seeds byproduct flour beverage−10% (UB10) and (**B**) extruded sesame seeds byproduct flour beverage-10 (EB10). 1: 3-*O*-p-Coumaroylquinic acid, 2: Vanillic acid, 3: Caffeic acid, 4: Chlorogenic acid, 5: Syringic acid; 6: Acteoside, 7: Ferulic Acid, 8: Diosmetin, 9: Verbasoside, 10: 6-Methylquercetin-3-*O*-Rutinoside, 11: Crenatoside, 12: Quercetin-3-*O*-glucoside, 13: Aspartic acid + 4(hexoside), 14: 2(3-Deoxyglucosone) + 2(γ-aminobutyric acid), 15: Luteolin-7-*O*-glucoside, 16: Isorhamnetin-7-*O*-glucoside, 17: Salvigenin, 18: Phlorizin, 19: Quercetin, 20: Luteolin, 21: Genistein-7-*O*-galactoside, 22: Apigenin, 23: Isorhamnetin, 24: Arginine + sucrose derived compound.

By analyzing the phytochemicals profile of sesame seeds byproduct beverages, 24 compounds (9 phenolic acids, 12 flavonoids, and 3 melanoidins) could be identified (Figure 2; Table 1). The extrusion process modified the phenolic profile of the beverage more qualitatively than quantitatively; seventeen of the 24 compounds were present in the unprocessed sesame seeds byproduct flour beverage−10% (UB10) and 21 in the extruded sesame seeds byproduct flour beverage−10% (EB10). The compounds caffeic acid, luteolin-7-*O*-glucoside, and isorhamnetin were identified only in UB10; while vanillic acid, acteoside, luteolin, quercetin, and melanoidins only in EB10. Except for isorhamnetin and melanoidins, all the compounds had been previously identified in sesame [16,17,33,34,35].

The extrusion process released the glucoside group (*m*/*z* = 162) of the compound luteolin-7-*O*-glucoside (*m*/*z* = 477.15) leaving the luteolin aglycone free (*m*/*z* = 285.04) in ESFB10. Similarly, the extrusion process broke the methyl group [*m*/*z* = 15] of the isorhamnetin (*m*/*z* = 315.11) generating quercetin (*m*/*z* = 300.99). A decrease in ferulic acid content was observed after the extrusion process (Table 1); this could be due to the transformation of ferulic acid to vanillic acid molecules, as the latter is an intermediate in forming vanillin from ferulic acid [38]. Ortega–Hernández et al. [39] report that in sesame, ferulic acid is found mainly in its free form, which makes it more susceptible to transformation or degradation due to some processes. Acteoside is a glucoside derived from hydroxytyrosol and caffeic acid [40]; thus, the presence of acteoside in EB10 explains the degradation of caffeic acid after extrusion. However, more specific studies are required to demonstrate that the extrusion process can form the ester and ether bonds between the hydroxytyrosol, caffeic acid, and glucoside compounds to generate the acteoside.

In addition, 3 new compounds resulting from the extrusion process were observed. These compounds had molecular ions of *m*/*z* = 780.24, *m*/*z* = 493.18, and *m*/*z* = 894.38. When this compounds were fragmented, they generated ions corresponding to saccharide molecules (*m*/*z* = 162.05, *m*/*z* = 162.14, and *m*/*z* = 341.3) and amino acids (*m*/*z* = 133.12, *m*/*z* = 103.50, and *m*/*z* = 174.5). These fragmentation patterns correspond to melanoidin-type compounds (Aspartic acid + 4(hexoside), 2(3-Deoxyglucosone) + 2(γ-aminobutyric acid), and Arginine + sucrose derived compound) generated by Maillard reactions [36,37]. When these compounds are produced in large quantities, can generate negative effects. However, the extrusion process is not drastic enough to produce these types of molecules in excess [41,42]. Recent studies have shown that in adequate concentrations, melanoidins generate health benefits [7,43]. Nevertheless, more specialized studies are needed to corroborate the identity, elucidate the structures, and know the bioactivity of extruded-sesame melanoidins.

Regarding the content of individual phenolic compounds, in Table 1 it can be seen that of all of the compounds identified, only ferulic acid showed statistically significant differences between UB10 and EB10. The most abundant phenolic acids were ferulic acid and chlorogenic acid for UB10 and EB10, respectively (Table 1). Ortega–Hernández et al. [39] report that the most abundant phenolic acid in sesame is ferulic acid, followed by protocatechuic acid and p-coumaric acid. On the contrary, Shahidi and Ambigaipalan [44] and Ghotbzadeh Kermani et al. [45] report caffeic acid as the majority in sesame seeds. El-Roby et al. [34] indicate that the most abundant flavonoids in sesame are catechin, followed by apigenin-7-*O*-glucoside and Chrysin. However, Morsy et al. [17] found that quercetin was the most abundant flavonoid. This latter was also the most abundant in EB10. Lin et al. [46] indicate that the sesame variety influences the presence of specific phenolic compounds and their concentration, as well other factors, such as soil fertility and sunlight.

In general, the extrusion process improved the phenolic compound profile of the beverage. The new phenolic compounds generated (vanillic acid, quercetin, and lutein), and some of those identified in both beverages (chlorogenic acid, ferulic acid, quercetin-3-*O*-glucoside, and isorhamnetin-7-*O*-glucoside); has shown to have good stability against the gastrointestinal digestion process and outstanding bioactivities [7,27,39,47,48,49,50]. However, it has also been observed that the bioaccessibility and bioactivity of phenolic compounds are a function of their concentration and the matrix from which they were extracted [47,50,51].

### 3.3. Total Phytochemical Content of UB10 and EB10

Despite the differences observed in the profile of phenolic compounds, no significant differences were observed in the total phytochemical content of UB10 and EB10 (*p* < 0.05) (Table 2). EB10 was shown to have good content of TPC and TF compared to a commercial beverage. The TPC values for UB10 and EB10 were 14.90 and 15.97 mg GAE/mL g of beverage. These values are higher than those observed by Nowak et al. [52] for the juice of elderberry, chokeberry, cranberry, wild rose, Japanese quince, sea buckthorn, and noni (6.82, 11.26, 2.77, 16.61, 11.08, 4.70, 3.00 mg GAE/100 mL). The TPC are lower than those reported by Behnam Nik and Vazifedoost [53] for a functional beverage of *Securigera securidaca* extract beverage (200 a 550 mg GAE/100 mL); this is partly due to the compounds used in the formulation of the beverage (Stevia, Honey, Apple Juice, citric acid, and mint) that by themselves contribute to TPC. Of the total TPC of sesame beverages, 38% correspond to TF. UB10 and EB10 were shown to have up to 1.5 times more TF than a commercial beverage. As in TPC, the extrusion process did not generate significant changes in the TF content of UB10 and EB10 (5.37 and 5.85 mg QE/100 mL of beverage) (*p* < 0.05). These values are similar to those reported by Nowak et al. [52] for the juice of different fruits (0.024 a 5.607 mg QE/100 mL). Elhanafi et al. [20] reported lower values than those observed in this study for beige sesame (10 mg QE/100 g). These differences could be due to differences in seed growing conditions and extraction methods. In this study, a hydroalcoholic extraction was made followed by acid hydrolysis, which could release a more significant number of compounds, while Elhanafi et al. [20] extracted only the free compounds.

### 3.4. Biological Activities

UB10 and EB10 showed higher biological activity than a commercial beverage (Table 3). Regarding to AA, EB10 showed better IC_50_ values than UB10. Park et al. [23] reported ABTS values of 15% of inhibition for seed sesame juice (1 mg/mL of extract). The values reported by these authors are lower than those observed in this study. On the other hand, Visavadiya et al. [19] reported IC_50_ values of 0.20 mg/mL of aqueous sesame extract by DPPH, similar to those obtained in this study. The differences in the AA are due to changes in the phenolic profile of beverages. Quintero–Soto et al. [27] reported that quercetin generates a higher AA than isorhamnetin. It has also been reported that Malliard reaction compounds generate greater AA than phenolic compounds [43]. This explains the higher AA values in EB10 and is consistent with the positive correlations observed in this research between these compounds and AA (Table 4).

UB10 and EB10 showed good inhibition of the *α*-amylase and *α*-glucosidase enzymes (Table 3). Both beverages more strongly inhibited the *α*-glucosidase enzyme than the *α*-amylase enzyme. Several researchers have observed this behavior in sesame [18,54]. This inhibition capacity is due to a higher affinity of the phenolics in beverages toward the *α*-glucosidase enzyme. For example, Mahnashi et al. [55] reported that ferulic, quercetin and quercetin-3-glucoside bind more strongly to *α*-glucosidase than to α-amylase, thus generating better inhibition.

EB10 showed a better value of IC_50_ (0.11 mg/mL) than UB10 (0.30 mg/mL) about DPP4 enzyme inhibition (Table 3). These values are similar to those reported by Meiliza et al. [56] for the infusion of *Camellia sinensis* (IC_50_ = 0.2 mg/mL) and lower than those observed by Amin et al. [57] for *Ipomoea batata* root extract (IC_50_ = 0.065 mg/mL). The higher DPP4 inhibition values generated by EB10 are due to the presence of specific phenolics [58]. A strong positive correlation was observed between the inhibition of DPP4 and the phenolics generated by the extrusion process (vanillic acid, acteoside, quercetin, and lutein); and a negative correlation between the compounds that were degraded (caffeic acid, lutein-7-*O*-glucoside, and isorhamnetin (Table 4).

### 3.5. Association between Phytochemicals Content and Biological Activities

**Figure 3:**
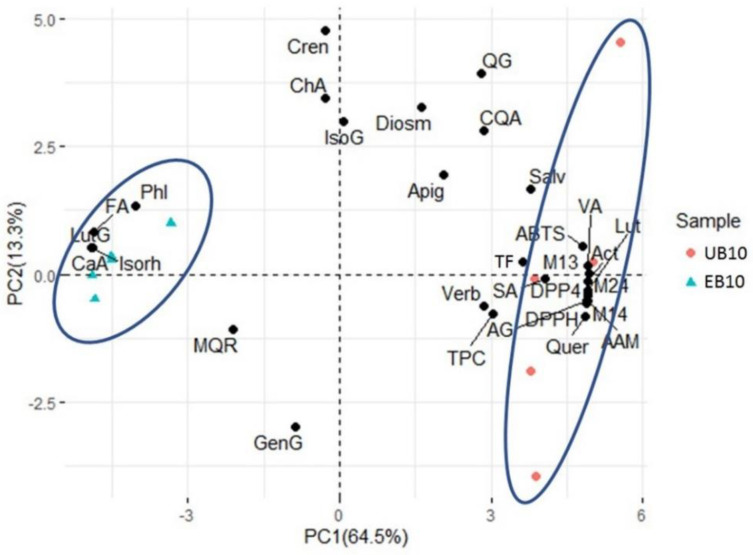
A biplot based on principal component analysis of metabolites and bioactivities. Samples are represented by colored-shapes and the variables by black points. CQA: 3-*O*-p-Coumaroylquinic acid, VA: Vanillic acid, CaA: Caffeic acid, ChA: Chlorogenic acid, SA: Syringic acid; Act: Acteoside, FA: Ferulic Acid, Diosm: Diosmetin, Verb: Verbasoside, MGR: 6-Methylquercetin-3-*O*-Rutinoside, Cren: Crenatoside, QG: Quercetin-3-*O*-glucoside, M13: Aspartic acid + 4(hexoside), M14: 2(3-Deoxyglucosone) + 2(γ-aminobutyric acid), LutG: Luteolin-7-*O*-glucoside, IsoG: Isorhamnetin-7-*O*-glucoside, Salv: Salvigenin, Phl: Phlorizin, Quer: Quercetin, Lut: Luteolin, GenG: Genistein-7-*O*-galactoside, Apig: Apigenin, Isorh: Isorhamnetin, M24: Arginine + sucrose derived compound, TPC: Total Phenolic Compounds: TF: Total Flavonoids, AAM: *α*-amylase inhibition AG: *α*-glucosidase inhibition, DPP4: Dipeptidyl-peptidase-IV inhibition.

Principal component analysis (PCA) was performed with the data of the content of phytochemical compounds and bioactivities (Figure 3). The two principal components explained 79% of the variation. Two clusters formed by the samples and variables studied were observed. Clusters of sesame metabolites were associated with the extrusion process.

The PCA showed a cluster in the negative quadrant of the principal component 1 (PC1) (left blue oval) formed by the samples UB10 and the metabolites caffeic acid, ferulic acid, 6-methylquercetin-3-*O*-rutinoside, phlorizin, luteolin, genistein -7-*O*-galactoside, and isorhamnetin. A second cluster was observed in the positive quadrant of PC1 (right blue oval) formed by samples EB10; the metabolites 3-*O*-p-coumaroylquinic acid, vanillic acid, syringic acid, acteoside, diosmetin, verbasoside, quercetin-3-*O*-glucoside, [aspartic acid + 4(hexoside)], [2(3-Deoxyglucosone) + 2(γ-aminobutyric acid)], salvigenin, luteolin, and [Arginine + sucrose derived compound]; and bioactivities (ABTS, DPPH, *α*-amylase inhibition, *α*-glucosidase inhibition, and DPP4 inhibition); indicating that the extrusion process had a significant positive impact on these variables (Figure 3). The EB10 samples were located in the right quadrants together with the majority of the metabolites, as well as the AA and enzyme inhibition; the correlation of these parameters supports the idea that the extrusion process is associated with a higher accumulation of antioxidant, hypoglycemic and antidiabetic metabolites, increasing the bioactivities of EB10.

## 4. Conclusions

This is the first research reporting the phenolic compounds profile of functional beverages based on sesame and their antidiabetic potential. In addition, it is the first report on identifying melanoidins (Maillard reaction compounds) in extruded sesame products. The extrusion process favorably modified the phytochemical profile of the beverage. The new phytochemical compounds generated by the extrusion process were strongly correlated with the bioactivities; therefore, they could be responsible. However, a synergistic effect between all the compounds is not ruled out. This research demonstrates the benefits of sesame seeds byproduct seed and the extrusion process to make a functional beverage with important bioactive compounds (phenolic acids, flavonoids, and melanoidins) and good antioxidant, hypoglycemic and antidiabetic properties. Sesame beverages could be an ideal vehicle to help improve the health of the people who consume them.

## Figures and Tables

**Table 1:** Phytochemical compounds found in extracts of unprocessed and extruded sesame seeds byproduct flour beverage.

#	[M-H]^-^	Main Fragment	Metabolite	Class	UB10 ^1^	EB10 ^1^	LOD	LOQ	Reference
1	337.11	191.05	3-O-*p*-Coumaroylquinic acid	Phenolic acid	12.25 ± 0.29 ^a^ (1.53 ± 0.04 ^a^)	13.00 ± 1.27 ^a^ (1.62 ± 0.16 ^a^)	0.0117	0.0355	[33]
2	167.09	108.06	Vanillic acid	Phenolic acid	ND	72.11 ± 11.93 (0.90 ± 0.15)	0.0048	0.0144	[33]
3	179.15	135.20	Caffeic acid	Phenolic acid	1.81 ± 0.12 (0.23 ± 0.01)	ND	0.0020	0.0060	[17,33]
4	353.05	191.01	Chlorogenic acid	Phenolic acid	25.03 ± 2.22 ^a^ (3.13 ± 0.28 ^a^)	24.82 ± 2.15 ^a^ (3.10 ± 0.27 ^a^)	0.0416	0.1259	[33]
5	197.05	123.00	Syringic acid	Phenolic acid	12.03 ± 1.12 ^a^ (1.50 ± 0.14 ^a^)	13.22 ± 0.07 ^a^ (1.65 ± 0.01 ^a^)	0.0034	0.0103	[16,17]
6	623.18	461.15	Acteoside	Phenolic acid	ND	1.03 ± 0.09 (0.13 ± 0.01)	0.0020	0.0060	[33]
7	193.05	175.02	Ferulic Acid	Phenolic acid	179.23 ± 4.71 ^a^ (22.40 ± 0.59 ^a^)	41.76 ± 8.03 ^b^ (5.22 ± 1.01 ^b^)	0.0090	0.0274	[17,33]
8	299.01	284.03	Diosmetin	Flavonoid	39.25 ± 3.53 ^a^ (4.90 ± 0.44 ^a^)	40.58 ± 3.97 ^a^ (5.07 ± 0.50 ^a^)	0.0057	0.0173	[33]
9	461.12	315.10	Verbasoside	Phenolic acid	0.47 ± 0.05 ^a^ (0.05 ± 0.01 ^a^)	0.52 ± 0.04 ^a^ (0.06 ± 0.01 ^a^)	0.0020	0.0060	[33]
10	624.01	317.06	6-Methylquercetin-3-*O*-Rutinoside	Flavonoid	17.75 ± 1.85 ^a^ (2.22 ± 0.23 ^a^)	17.07 ± 1.08 ^a^ (2.13 ± 0.20 ^a^)	0.0003	0.0011	[33]
11	621.18	162.00	Crenatoside	Phenolic acid	0.67 ± 0.02 ^a^ (0.08 ± 0.01 ^a^)	0.66 ± 0.07 ^a^ (0.08 ± 0.01 ^a^)	0.0020	0.0060	[33]
12	463.12	300.05	Quercetin-3-*O*-glucoside	Flavonoid	36.10 ± 0.16 ^a^ (4.51 ± 0.02 ^a^)	36.94 ± 1.44 ^a^ (4.62 ± 0.18 ^a^)	0.0020	0.0061	[33]
13	780.24	647.12	Aspartic acid + 4(hexoside)	Melanoidin	ND	NQ	-	-	[36]
14	493.18	385.05	2(3-Deoxyglucosone) + 2(γ-aminobutyric acid)	Melanoidin	ND	NQ	-	-	[37]
15	447.15	285.08	Luteolin-7-*O*-glucoside	Flavonoid	18.42 ± 0.48 (2.30 ± 0.06)	ND	0.0024	0.0071	[33]
16	477.11	315.18	Isorhamnetin-7-*O*-glucoside	Flavonoid	679.41 ± 27.96 ^a^ (84.93 ± 3.50 ^a^)	683.71 ± 27.85 ^a^ (85.49 ± 3.48 ^a^)	0.0779	0.2362	[33]
17	327.09	294.02	Salvigenin	Flavonoid	159.40 ± 7.86 ^a^ (19.92 ± 0.98 ^a^)	169.68 ± 0.62 ^a^ (21.21 ± 1.20 ^a^)	0.0057	0.0173	[33]
18	435.13	166.65	Phlorizin	Flavonoid	88.60 ± 0.75 ^a^ (11.07 ± 0.75 ^a^)	79.27 ± 5.00 ^a^ (9.90 ± 0.63 ^a^)	0.0024	0.0071	[33]
19	300.09	179.01	Quercetin	Flavonoid	ND	867.57 ± 63.89 (108.45 ± 7.99)	0.0020	0.0061	[17,27]
20	285.04	151.00	Luteolin	Flavonoid	ND	26.61 ± 1.38 (3.33 ± 0.17)	0.0024	0.0071	[33]
21	430.95	269.22	Genistein-7-*O*-galactoside	Flavonoid	9.47 ± 0.98 ^a^ (1.18 ± 0.12 ^a^)	9.11 ± 1.34 ^a^ (1.14 ± 0.17 ^a^)	0.0058	0.0175	[33]
22	269.22	153.02	Apigenin	Flavonoid	15.76 ± 1.05 ^a^ (1.97 ± 0.13 ^a^)	16.64 ± 1.49 ^a^ (2.08 ± 0.19 ^a^)	0.0058	0.0175	[16]
23	315.11	300.05	Isorhamnetin	Flavonoid	1559.41 ± 35.77 (95.45 ± 2.17)	ND	0.0779	0.2362	[27]
24	894.38	683.25	Arginine + sucrose derived compound	Melanoidin	ND	NQ	-	-	[36]

LOD = Limit of detection (mg/mL); LOQ = Limit of quantification (mg/mL). ND = Not Detected; NQ = Not quantified. UB10 = Unprocessed sesame seeds byproduct flour beverage−10%; EB10 = Extruded sesame seeds byproduct flour beverage−10%; The number 10 in UB10 and EB10 indicates the concentration of raw/extruded SSBPF used in the preparation of the beverage. ^1^ The results are expressed in mg/100 g dw and mg/100 mL of beverage (values in parentheses). Different letters in the same line indicate significant differences (*p* < 0.05) among the means according to the Fisher’s test.

**Table 2:** Total phytochemical content of unprocessed and extruded sesame seeds byproduct flour beverages, and commercial beverage.

Feature	UB10	EB10	Commercial
**Phenolic Compounds**			
(mg GAE/100 g dw)	114.19 ± 5.67 ^a^	123.15 ± 7.50 ^a^	90.15 ± 5.32 ^b^
(mg GAE/100 mL beverage)	14.90 ± 0.71 ^a^	15.97 ± 1.00 ^a^	5.11 ± 0.22 ^b^
**Flavonoids**			
(mg QE/100 g dw)	43.80 ± 3.75 ^a^	47.90 ± 1.60 ^a^	30.25 ± 2.75 ^b^
(mg QE/100 mL beverage)	5.37 ± 0.47 ^a^	5.85 ± 0.30 ^a^	2.22 ± 0.90 ^b^

UB10 = Unprocessed sesame seeds byproduct flour beverage−10%; EB10 = Extruded sesame seeds byproduct flour beverage−10%; GAE: gallic acid equivalent. QE: quercetin equivalent. dw: dry weight. Different letters in the same row indicate significant differences (*p* < 0.05) between the means according to Fisher’s test.

**Table 3:** Antioxidant activity, hypoglycemic activity, and antidiabetic activity of unprocessed and extruded sesame seeds byproduct flour beverages, and commercial beverage.

Feature	UB10	EB10	Commercial	Positive Control *
**Antioxidant activity (AA)**				
**ABTS**				
(µmol TE/100 g dw)	1668.61 ± 69.91 ^b^	2010.98 ± 57.85 ^a^	660.99 ± 15.89 ^c^	
(µmol TE/100 mL beverage)	208.57 ± 8.73 ^b^	251.30 ± 7.21 ^a^	120.00 ± 9.12 ^c^	
IC_50_ (mg/mL extract)	0.24 ± 0.02 ^b^	0.19 ± 0.01 ^c^	0.52 ± 0.02 ^a^	0.03 ± 0.00
**DPPH**				
(µmol TE/100 g dw)	744.92 ± 30.52 ^b^	1770.65 ± 37.73 ^a^	450.12 ± 12.77 ^c^	
(µmol TE/100mL beverage)	93.12 ± 3.81 ^b^	221.27 ± 4.68 ^a^	55.01 ± 5.28 ^c^	
IC_50_ (mg/mL extract)	0.31 ± 0.01 ^b^	0.21 ± 0.01 ^c^	0.92 ± 0.03 ^a^	0.02 ± 0.00
**Hypoglycemic activity**				
**Inhibition of *α*-amylase** IC_50_ (mg/mL extract)	2.29 ± 0.03 ^b^	1.01 ± 0.01 ^c^	7.21 ± 0.15 ^a^	0.06 ± 0.00
% of inhibition of/100 mL beverage	52.32 ± 0.61 ^b^	95.75 ± 0.40 ^a^	12.13 ± 0.34 ^c^	
**Inhibition of *α*-glucosidase** IC_50_ (mg/mL extract)	0.47 ± 0.01 ^b^	0.17 ± 0.01 ^c^	3.11 ± 0.21 ^a^	3.98 ± 0.09
% of inhibition of/100 mL beverage	>100	>100	28.20 ± 2.00	
**Antidiabetic activity**				
**DPP4 inhibition** IC_50_ (mg/mL extract)	0.30 ± 0.01 ^b^	0.11 ± 0.01 ^c^	0.81 ± 0.01 ^a^	0.01 ± 0.00
% of inhibition of/100 mL beverage	>100	>100	81.25 ± 0.75	

UB10 = Unprocessed sesame seeds byproduct flour beverage−10%; EB10 = Extruded sesame seeds byproduct flour beverage−10%; TE: trolox equivalent. dw: dry weight. * Positive control IC_50_ values were expressed in mg of trolox/ml for ABTS and DPPH inhibition, mg of acarbose/ ml for α-amylase and α-glucosidase inhibition, and mg of sitagliptin/mL for DPP4 inhibition. Different letters in the same row indicate significant differences (*p* < 0.05) between the means according to Fisher’s test.

**Table 4:** Correlation of metabolites and bioactivities.

	CQA	VA	CaA	ChA	SA	Act	FA	Diosm	Verb	MQR	Cren	QG	M13	M14	LutG
**CQA**	1														
**VA**	NS	1													
**CaA**	NS	−0.985 ***	1												
**ChA**	NS	NS	NS	1											
**SA**	NS	0.765 **	−0.801 **	NS	1										
**Act**	NS	0.997 ***	−0.994 ***	NS	0.774 **	1									
**FA**	NS	−0.977 ***	0.995 ***	NS	−0.764 **	−0.989 ***	1								
**Diosm**	NS	NS	NS	NS	NS	NS	NS	1							
**Verb**	NS	NS	−0.652 *	NS	NS	0.640 *	−0.661 *	NS	1						
**MQR**	NS	NS	NS	NS	NS	NS	NS	NS	NS	1					
**Cren**	NS	NS	NS	NS	NS	NS	NS	NS	NS	NS	1				
**QG**	0.871 **	NS	NS	NS	NS	NS	NS	NS	NS	NS	0.751 *	1			
**M13**	NS	0.999 ***	−0.990 ***	NS	0.770 **	0.999 ***	−0.983 ***	NS	NS	NS	NS	NS	1		
**M14**	NS	0.991 ***	−0.997 ***	NS	0.779 **	0.997 ***	−0.996 ***	NS	NS	NS	NS	NS	0.994 ***	1	
**LutG**	NS	−0.987 ***	0.999 ***	NS	−0.796 **	−0.996 ***	0.997 ***	NS	−0.645 *	NS	NS	NS	−0.991 ***	−0.998 ***	1
**IsoG**	NS	NS	NS	NS	NS	NS	NS	NS	NS	NS	NS	NS	NS	NS	NS
**Salv**	NS	0.756 ***	−0.701 *	NS	0.775 **	0.721 *	−0.647 *	NS	NS	−0.771 **	NS	0.752 *	0.743 *	0.709 *	−0.690 *
**Phl**	NS	−0.744 **	0.791 **	NS	−0.830 **	−0.783 **	0.807 **	NS	NS	0.702 *	NS	NS	−0.780 **	−0.811 **	0.804 **
**Quer**	NS	0.979 ***	−0.996 ***	NS	0.781 **	0.990 ***	−0.999 ***	NS	0.636 *	NS	NS	NS	0.985 ***	0.998 ***	−0.997 ***
**Lut**	NS	0.994 ***	−0.997 ***	NS	0.778 **	0.999 ***	−0.994 ***	NS	0.647 *	NS	NS	NS	0.997 ***	0.999 ***	−0.998 ***
**GenG**	NS	NS	NS	NS	NS	NS	NS	NS	NS	NS	−0.755 *	NS	NS	NS	NS
**Apig**	NS	NS	NS	NS	NS	NS	NS	NS	0.712 *	NS	NS	NS	NS	NS	NS
**Isorh**	NS	−0.987 ***	0.999 ***	NS	−0.794 **	−0.996 ***	0.997 ***	NS	0.645 *	NS	NS	NS	−0.991 ***	−0.998 ***	0.999 ***
**M24**	NS	0.992 ***	−0.997 ***	NS	0.778 **	0.999 ***	−0.995 ***	NS	0.658 *	NS	NS	NS	0.996 ***	0.997 ***	−0.999 ***
**ABTS**	NS	0.958 ***	−0.951 ***	NS	0.873 ***	0.958 ***	−0.931 ***	NS	NS	NS	NS	NS	0.958 ***	0.945 ***	0.953 ***
**DPPH**	NS	0.985 ***	−0.996 ***	NS	0.779 **	0.994 ***	−0.997 ***	NS	0.651 *	NS	NS	NS	NS	0.997 ***	0.998 ***
**TPC**	NS	NS	NS	NS	NS	NS	NS	NS	NS	NS	NS	NS	NS	NS	NS
**TF**	NS	0.706 *	−0.672 *	NS	NS	0.700 *	−0.686 *	NS	NS	NS	NS	NS	0.704 *	0.696 *	−0.690 *
**AAM**	NS	0.987 ***	−0.997 ***	NS	0.785 **	0.996 ***	−0.997 ***	NS	0.644 *	NS	NS	NS	0.992 ***	0.998 ***	−0.999 ***
**AG**	NS	0.987 ***	−0.999 ***	NS	0.778 **	0.996 ***	−0.997 ***	NS	0.663 *	NS	NS	NS	0.992 ***	0.998 ***	−0.999 ***
**DPP4**	NS	0.992 ***	−0.998 ***	NS	0.772 **	0.998 ***	−0.996 ***	NS	0.654 *	NS	NS	NS	0.995 ***	0.999 ***	−0.999 ***
	**IsoG**	**Salv**	**Phl**	**Quer**	**Lut**	**GenG**	**Apig**	**Isorh**	**M24**	**ABTS**	**DPH**	**TPC**	**TF**	**AAM**	**AG**
**CQA**															
**VA**															
**CaA**															
**ChA**															
**SA**															
**Act**															
**FA**															
**Diosm**															
**Verb**															
**MQR**															
**Cren**															
**QG**															
**M13**															
**M14**															
**LutG**															
**IsoG**	1														
**Salv**	NS	1													
**Phl**	NS	−0.664 *	1												
**Quer**	NS	0.676 *	−0.819 **	1											
**Lut**	NS	NS	−0.791 **	0.995	1										
**GenG**	NS	NS	NS	NS	NS	1									
**Apig**	NS	NS	NS	NS	NS	−0.927 ***	1								
**Isorh**	NS	−0.688 *	0.806 **	−0.998 ***	−0.998 ***	NS	NS	1							
**M24**	NS	0.694 *	−0.786 **	0.994 ***	0.999 ***	NS	NS	−0.999 ***	1						
**ABTS**	NS	0.764 **	−0.793 **	0.934 ***	0.954 ***	NS	NS	−0.953 ***	0.955 ***	1					
**DPPH**	NS	0.680 *	−0.801 **	0.996 ***	0.997 ***	NS	NS	−0.998 ***	0.997 ***	0.943 ***	1				
**TPC**	NS	NS	−0.715 *	NS	NS	NS	NS	NS	NS	NS	NS	1			
**TF**	NS	NS	−0.735 *	0.685 *	0.696 *	NS	NS	−0.693 *	0.693 *	0.719 *	0.661 *	0.764 **	1		
**AAM**	NS	0.680 *	−0.809 **	0.997 ***	0.998 ***	NS	NS	1	0.999 ***	0.953 ***	0.998 ***	NS	0.703 *	1	
**AG**	NS	0.687 *	−0.788 **	0.997 ***	0.999 ***	NS	NS	−0.999 ***	0.999 ***	0.946 ***	0.988 ***	NS	0.679*	0.998 ***	1
**DPP4**	NS	0.698 *	−0.789 **	0.996 ***	0.999 ***	NS	NS	−0.999 ***	0.999 ***	0.948 ***	0.997 ***	NS	0.689*	0.998 ***	0.999 ***

NS: represent nonsignificant coefficients. *** Correlation is significant at the 0.001 level, ** Correlation is significant at the 0.01 level, * Correlation is significant at the 0.05 level. CQA: 3-*O*-p-Coumaroylquinic acid, VA: Vanillic acid, CaA: Caffeic acid, ChA: Chlorogenic acid, SA: Syringic acid; Act: Acteoside, FA: Ferulic Acid, Diosm: Diosmetin, Verb: Verbasoside, MGR: 6-Methylquercetin-3-*O*-Rutinoside, Cren: Crenatoside, QG: Quercetin-3-*O*-glucoside, M13: Aspartic acid + 4(hexoside), M14: 2(3-Deoxyglucosone) + 2(γ-aminobutyric acid), LutG: Luteolin-7-*O*-glucoside, IsoG: Isorhamnetin-7-*O*-glucoside, Salv: Salvigenin, Phl: Phlorizin, Quer: Quercetin, Lut: Luteolin, GenG: Genistein-7-*O*-galactoside, Apig: Apigenin, Isorh: Isorhamnetin, M24: Arginine + sucrose derived compound, TPC: Total Phenolic Compounds: TF: Total Flavonoids, AAM: *α*-amylase inhibition AG: *α*-glucosidase inhibition, DPP4: Dipeptidyl-peptidase-IV inhibition.

## Data Availability

The datasets generated and/or analyzed during the current study are available from the corresponding authors on reasonable request.
